# Inflammatory Fibroid Polyp of the Small Intestine Presenting With Intussusception and Secondary Appendicitis, Successfully Treated Endoscopically

**DOI:** 10.1016/j.gastha.2025.100878

**Published:** 2025-12-31

**Authors:** Kazuki Natsui, Manabu Takeuchi, Shuji Terai

**Affiliations:** 1 Department of Gastroenterology, Nagaoka Red Cross Hospital, Nagaoka, Niigata, Japan; 2 Division of Gastroenterology and Hepatology, Graduate School of Medical and Dental Sciences, Niigata University, Niigata, Japan

A 53-year-old man presented with several days of worsening abdominal pain and diarrhea. Physical examination showed right lower quadrant rebound tenderness. Computed tomography revealed a 3.5 cm-sized pedunculated lesion originating from the terminal ileum, invaginating into the ascending colon and causing intussusception and secondary appendicitis (Figure A). As no intestinal ischemia was observed, conservative treatment was administered, and the patient recovered. Colonoscopy performed after discharge revealed enlargement of the ileocecal valve and a reddish pedunculated lesion located 10 cm proximal to the valve, with a granular surface and erosions at the top (Figure B). Biopsy specimens revealed nonneoplastic small intestinal epithelium; therefore, diagnostic and therapeutic endoscopic resection was performed. Snare polypectomy with prevention-of-bleeding clipping was successfully completed. The lesion measured 32 × 22 × 45 mm (Figure C). Histopathological examination showed submucosal proliferation of blood vessels and inflammatory cells, predominantly eosinophils, leading to a diagnosis of inflammatory fibroid polyp (IFP; Figure D). The lesion was completely resected, and the patient was discharged without complications.

**Figure 1:**
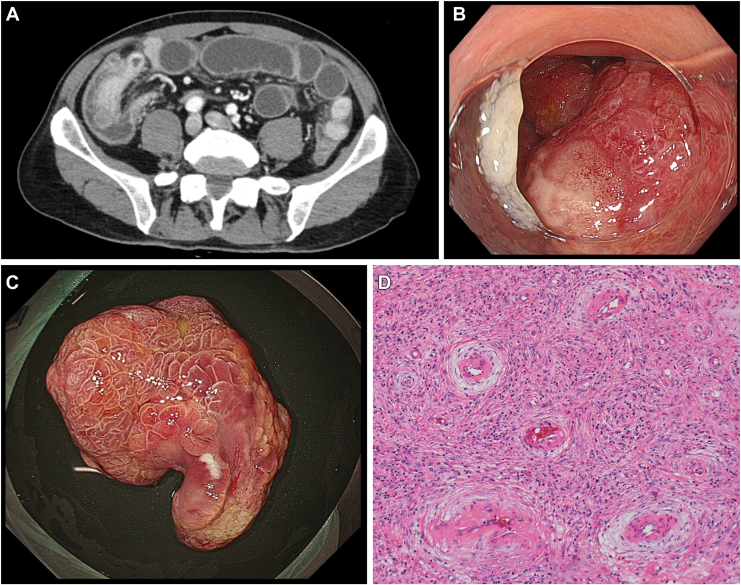

IFP is a rare benign submucosal lesion of the gastrointestinal tract that may cause intussusception or bleeding, particularly in the small intestine. IFP should be considered in the differential diagnosis of small intestinal lesions causing intussusception.

